# Tobacco and Cannabis Co-use by HIV Status Among United States Adults: Results from the 2021–2023 National Survey on Drug Use and Health

**DOI:** 10.1007/s10461-025-04817-5

**Published:** 2025-07-04

**Authors:** Juhan Lee, Ana Paula Xingru Yin, Andrea H. Weinberger

**Affiliations:** 1 Department of Epidemiology and Population Health, Albert Einstein College of Medicine, Van Etten Building, Room 3A02F, 1225 Morris Park Ave, Bronx, NY 10451 USA; 2 Department of Epidemiology and Population Health, Montefiore Einstein Comprehensive Cancer Center, New York, USA; 3 Ferkauf Graduate School of Psychology, Yeshiva University, New York, USA; 4 Department of Psychiatry & Behavioral Sciences, Albert Einstein College of Medicine, Bronx, USA

**Keywords:** HIV, Tobacco, Smoking, Cannabis

## Abstract

Tobacco and cannabis use are prevalent among adults with HIV and co-use is associated with drug dependence and negative health outcomes such as lung disease. This study examined the co-use of tobacco and cannabis by HIV status overall and by sociodemographics. Data came from the adult sample from the 2021–2023 National Survey on Drug Use and Health. Adjusted multinomial logistic regressions were performed on tobacco cannabis co-use by HIV status, adjusting for covariates. We tested potential interaction effects between HIV status and each sociodemographic factor (i.e., age, sex, race/ethnicity, income, state-level medical cannabis legalization status) on the outcome. Among total adult respondents (*N* = 139,524), 0.4% (weighted) reported lifetime HIV diagnosis and 8.2% reported past-month tobacco and cannabis co-use. After adjusting for covariates, adults with HIV, compared with adults without HIV, were more likely to report past-month tobacco and cannabis co-use (aRRR = 3.35, 95% CI = 1.79, 6.27). We also observed that race/ethnicity and state-level medical cannabis legalization status modified the association between HIV diagnosis and tobacco and cannabis co-use. This study observed a higher prevalence of tobacco and cannabis co-use among adults with HIV overall and identified subgroup sociodemographic differences by race/ethnicity and state-level medical cannabis legalization.

**Supplementary Information:**

The online version contains supplementary material available at 10.1007/s10461-025-04817-5.

## Background

Co-use of tobacco and cannabis has significantly increased among the general population in the United States (U.S.) [1], with 8.5% of U.S. adults reporting past-month tobacco and cannabis co-use in 2022 [2]. The co-use of tobacco and cannabis is associated with increased risks of heavy use of tobacco and cannabis, difficulty quitting tobacco and cannabis, and chronic illnesses (e.g., pulmonary function impairment) [3, 4]. Concerningly, the co-use of tobacco and cannabis might be also common among people with chronic diseases such as Human Immunodeficiency Virus (HIV). Tobacco use is common among individuals with HIV, with over 30% reporting tobacco use, including cigarette smoking [5–9], significantly increasing risks of cancer, cardiovascular diseases (e.g., stroke), pulmonary diseases, lung infections, and other inflammatory conditions, as well as reduced life expectancy [10, 11]. Cannabis use is also prevalent among people with HIV, with over 30% reporting past-year use [12–14]. While some evidence suggests therapeutic effects of medicinal cannabis use among people with HIV, such as alleviating symptoms, pain management, and anti-inflammatory effects [14], other studies indicate potential adverse effects, including an increased risk of pulmonary diseases, infections, and neurocognitive impairment (e.g., memory deficits) [13, 15].

According to the U.S. Department of Health & Human Services, approximately 1.2 million people aged 13 and older were living with HIV in the U.S. as of February 2025 [16]. Given that tobacco and cannabis co-use is known to exacerbate health risks and use dependence [17, 18], co-use may similarly negatively affect health outcomes among people with HIV. For example, Lorenz et al. reported higher incidence rates of lung infections (e.g., influenza, viral pneumonia, other pneumonia) among people with HIV who used both cannabis and tobacco compared to people with HIV with no cannabis use or with exclusive cannabis use [19]. Tobacco and cannabis co-use may also negatively impact the cessation of either product. Cannabis use is associated with lower odds of a cigarette quit attempt and former cigarette smoking status among people with HIV [20–22]. Further, Ozga et al. analyzed data from a randomized controlled trial for cigarette smoking cessation among treatment-seeking people with HIV and found that increased cannabis use between baseline and follow-up (compared to no change or decreased cannabis use) was associated with a lower likelihood of cigarette smoking cessation [23]. Understanding the patterns and effects of tobacco and cannabis co-use among people with HIV is critical, as co-use may further compromise health outcomes. Such understanding can inform the development of tailored prevention and cessation strategies for individuals with HIV.

Several key knowledge gaps remain regarding the co-use of tobacco and cannabis by HIV status at the U.S. population level. First, previous studies of tobacco and cannabis use among people with HIV have primarily analyzed data from randomized controlled trials which may not be generalizable to the broader U.S. population [23], lacked comparisons with people without HIV [23], or have relied on older data that may not reflect the range of current tobacco and cannabis products or changes in the legalization status of cannabis [19]. Second, research is needed to explore how tobacco and cannabis co-use varies by sociodemographic factors, as both HIV and substance use differs by variables such as age, sex, race/ethnicity, and income level. For example, young adults (aged 25–34), males, Black and Hispanic individuals, and people with lower income demonstrate both a higher prevalence and incidence of HIV, as well as a high prevalence of substance use when compared to older adults, females, White and non-Hispanic individuals, and people with higher income, respectively [24, 25]. In addition, living in a state with a medical cannabis law is associated with greater tobacco and cannabis co-use in non-HIV-specific samples [26]. A comprehensive understanding of tobacco and cannabis co-use prevalence by HIV status, overall and by these key sociodemographic variables, is critical for developing targeted prevention and cessation strategies tailored to this population.

The overall aim of this study was to use data from a nationally representative U.S. adult sample to examine tobacco and cannabis co-use by HIV status. First, we examined the prevalence of tobacco and cannabis co-use by HIV status, hypothesizing that individuals with HIV would be more likely to report tobacco and cannabis co-use compared to individuals without HIV. Second, we explored sociodemographic differences in tobacco and cannabis co-use patterns, hypothesizing that age, sex, race/ethnicity, annual income, and state-level medical cannabis legalization status would modify the association of HIV diagnosis and tobacco and cannabis co-use.

## Methods

### Dataset and Study Population

We utilized the adult dataset from the 2021–2023 National Survey on Drug Use and Health (NSDUH), a nationally representative survey conducted by the U.S. Substance Abuse and Mental Health Services Administration. The NSDUH employs a multilevel stratified sampling design to survey civilian, non-institutionalized individuals in the U.S. Its complex sampling design and weighting allow for national estimates based on survey responses. This study included all adult respondents from the pooled 2021–2023 dataset (*N* = 139,524).

### Measures

The primary outcome was past-month tobacco and cannabis co-use, categorized into four groups: no tobacco or cannabis use, tobacco use only, cannabis use only, and co-use of tobacco and cannabis. Tobacco use included self-reported use of any cigarettes, nicotine vaping products, cigars, pipes, or smokeless tobacco in the past month. Cannabis use included self-reported use of any form of cannabis consumption in the past month, including smoking, vaping, dabbing cannabis waxes, shatter, or concentrates; ingestion via edibles or drinks; oral use of cannabis drops, strips, lozenges, or sprays; topical application of cannabis lotions, creams, or patches; and cannabis pills. The primary predictor was self-reported lifetime HIV or AIDS diagnosis, assessed by the question: “Has a doctor or other healthcare professional ever told you that you had HIV or AIDS?” (no, yes).

 Covariates were selected based on existing literature on factors associated with tobacco and cannabis co-use [ 2, 26, 27 ], while ensuring no collinearity with lifetime HIV diagnosis. These included: age (young adults [18–25 years], other adults [≥ 26 years]), sex (female, male), race/ethnicity (non-Hispanic White, non-Hispanic Black, Hispanic, non-Hispanic other), annual income (<$75,000, ≥$75,000), state-level medical cannabis legalization (no, yes), and survey year (2021, 2022, 2023). Age groups were defined as “young adults” and “other adults,” as young adults are more likely to report tobacco and cannabis co-use [ 27, 28 ], and income brackets were defined according to the median household income in the U.S. in 2022 ($75,000) [ 29 ].

### Statistical Analysis

We first conducted descriptive analyses among all adult respondents from the 2021–2023 dataset, stratified by tobacco and cannabis co-use status. Rao-Scott adjusted Pearson chi-squared tests were used to examine bivariate associations between co-use status, lifetime HIV diagnosis, and other covariates.

We then conducted an adjusted multinomial logistic regression to examine the association between tobacco and cannabis co-use and lifetime HIV diagnosis, adjusting for all listed covariates. As sensitivity analyses, we re-ran multinomial logistic regression models incorporating each additional covariate: (1) sexual identity (heterosexual/straight, gay/lesbian, bisexual), (2) past-month use of other substances (none versus any; including alcohol, cocaine/crack, heroin, hallucinogens, inhalants, methamphetamine, and misuse of pain relievers, tranquilizers, stimulants, or sedatives), and (3) past-month serious psychological distress (assessed with Kessler-6 [30]; K6 score ≥ 13 as a threshold for serious psychological distress; no versus yes) (Supplemental Tables 1–3). These variables were excluded from the primary models since they were significantly associated with HIV status based on the previous literature and bivariate analyses [31–35].

Further, we conducted the adjusted multinomial logistic regression to examine the main and interaction effects between HIV diagnosis and sociodemographic factors on tobacco and cannabis co-use. Separate multinomial logistic regression models tested interactions: (1) x age group, (2) x sex, (3) x race/ethnicity, (4) x annual income, and (5) x state-level medical cannabis legalization status. For each analysis, we included an interaction term between HIV diagnosis and the moderator variable while adjusting for all listed covariates. For example, to test age as a moderator, we modeled co-use as a function of HIV diagnosis, age group, and their interaction term. After running these models, we calculated the linear combination of coefficients of the main effect of HIV status and the interaction effect between HIV status and each moderator variable.

All analyses accounted for the NSDUH complex sampling design and applied survey weights. Following NSDUH guidelines, a new sampling weight was created by dividing the provided weight by the number of pooled years [36]. We used a complete-case approach using the logistic regression model’s maximum likelihood estimation, resulting in a final analytic sample of *N* = 135,638. Missing data accounted for 2.78% of cases, a level unlikely to bias results [37]. Statistical significance was set at *p* < 0.05 (two-tailed). This study followed the Strengthening the Reporting of Observational Studies in Epidemiology (STROBE) guidelines. Secondary analysis of de-identified, publicly available U.S. national data is exempt from Institutional Review Board review. All analyses were conducted using STATA 18.0 (College Station, TX).

## Results

 Table  1 presents the sample characteristics of all adult respondents and stratifies them by tobacco and cannabis co-use status in the 2021–2023 NSDUH dataset ( *N*  = 139,524). Among all adult respondents, 13.3% were young adults (18–25 years), 51.3% were female, 12.1% were non-Hispanic Black, 17.3% were Hispanic, 57.7% had an annual income below $75,000, and 72.9% resided in states with legalized medical cannabis. Regarding substance use patterns, 16.1% (weighted) reported past-month tobacco use only, 7.3% reported past-month cannabis use only, and 8.2% reported past-month tobacco and cannabis co-use. Lifetime HIV diagnosis was reported by 0.4% of respondents, representing an estimated 911,571 U.S. adults in 2021–2023. Tobacco and cannabis co-use among adults with HIV was estimated at 178,775 U.S. adults.

**Table 1:** Sample characteristics by tobacco and cannabis co-use status, among adults from 2021–2023 NSDUH

	Total Sample	No Tobacco or Cannabis Use	Tobacco Only Use	Cannabis Only Use	Co-use of tobacco and cannabis
	139,524 (100)	92,525 (68.4)	21,288 (16.1)	11,809 (7.3)	13,902 (8.2)
Lifetime HIV diagnosis status					
No	135,302 (99.6)	90,067 (68.6)	20,477 (16.1)	11,422 (7.3)	13,336 (8.1)
Yes	336 (0.4)	134 (40.7)	72 (21.9)	59 (17.8)	71 (19.6)
Survey years					
2021	47,291 (33.1)	32,556 (69.3)	6,881 (16.9)	3,759 (6.3)	4,095 (7.6)
2022	47,100 (33.4)	30,988 (68.2)	7,256 (15.8)	3,944 (7.5)	4,912 (8.5)
2023	45,133 (33.5)	28,981 (67.8)	7,151 (15.7)	4,106 (8.0)	4,895 (8.4)
Age, years					
26 or older	97,651 (86.7)	66,840 (69.5)	15,421 (16.5)	7,621 (6.8)	7,769 (7.1)
18–25	41,873 (13.3)	25,685 (61.3)	5,867 (13.5)	4,188 (10.0)	6,133 (15.2)
Sex					
Female	77,598 (51.3)	54,667 (73.7)	10,048 (13.3)	6,483 (6.8)	6,400 (6.3)
Male	61,926 (48.7)	37,858 (62.9)	11,240 (19.2)	5,326 (7.8)	7,502 (10.2)
Race/ethnicity					
Non-Hispanic White	83,829 (61.7)	54,438 (65.9)	13,915 (18.0)	7,231 (7.6)	8,245 (8.5)
Non-Hispanic Black	15,883 (12.1)	10,190 (66.8)	2,342 (15.6)	1,404 (7.5)	1,947 (10.1)
Hispanic	24,394 (17.3)	17,314 (74.8)	2,904 (12.1)	2,076 (6.7)	2,100 (6.4)
Non-Hispanic Other Races	15,418 (8.9)	10,583 (75.8)	2,127 (11.7)	1,098 (5.5)	1,610 (7.0)
Annual income level					
Less than $75,000	82,653 (57.7)	50,670 (64.0)	14,742 (19.1)	6,921 (6.8)	10,320 (10.1)
$75,000 or more	56,871 (42.3)	41,855 (74.5)	6,546 (12.1)	4,888 (7.9)	3,582 (5.6)
Living in a state with a medical cannabis law					
No	33,743 (27.1)	22,723 (68.3)	6,269 (20.0)	1,861 (4.6)	2,890 (7.1)
Yes	105,781 (72.9)	69,802 (68.5)	15,019 (14.7)	9,948 (8.3)	11,012 (8.6)

Key: NSDUH = National Survey on Drug Use and Health

Unweighted n and row weighted % indicated

Table 2 presents the results of the adjusted multinomial logistic regression examining the association between past-month tobacco and cannabis use and lifetime HIV diagnosis. After adjusting for covariates, individuals with a lifetime HIV diagnosis were more likely to report past-month tobacco only use (adjusted relative risk ratio [aRRR] = 1.87, 95% CI: 1.07–3.26), past-month cannabis only use (aRRR = 3.97, 95% CI: 2.25–7.01), and past-month tobacco and cannabis co-use (aRRR = 3.35, 95% CI: 1.79–6.27) compared to individuals without HIV. Supplemental Tables 1–3 present the results of sensitivity analyses for an adjusted multinomial logistic regression model on the tobacco and cannabis co-use by HIV status, adjusting for each additional covariate. Although the magnitude was variable, similar significance was found across all three models, indicating that U.S. adults with HIV consistently reported higher levels of tobacco and cannabis co-use compared to those without HIV (aRRR range: 2.10–3.33).

**Table 2:** Results of adjusted multinomial logistic regression on tobacco and cannabis co-use by lifetime HIV diagnosis status, among adults from 2021–2023 NSDUH

	Tobacco only use		Cannabis only use		Co-use of tobacco and cannabis	
	aRRR (95% CI)	p-value	aRRR (95% CI)	p-value	aRRR (95% CI)	p-value
Lifetime HIV diagnosis status						
No	Reference		Reference		Reference	
Yes	1.87 (1.07, 3.26)	0.029	3.97 (2.25, 7.01)	< 0.001	3.35 (1.79, 6.27)	< 0.001
Survey years						
2021	Reference		Reference		Reference	
2022	0.98 (0.91, 1.06)	0.573	1.20 (1.07, 1.36)	0.003	1.16 (1.07, 1.26)	< 0.001
2023	1.00 (0.93, 1.08)	0.989	1.31 (1.17, 1.47)	< 0.001	1.20 (1.09, 1.33)	0.001
Age, years						
26 or older	Reference		Reference		Reference	
18–25	0.92 (0.86, 0.98)	0.017	1.74 (1.64, 1.86)	< 0.001	2.41 (2.22, 2.62)	< 0.001
Sex						
Female	Reference		Reference		Reference	
Male	1.77 (1.68, 1.88)	< 0.001	1.34 (1.23, 1.44)	< 0.001	2.07 (1.95, 2.19)	< 0.001
Race/ethnicity						
Non-Hispanic White	Reference		Reference		Reference	
Non-Hispanic Black	0.73 (0.68, 0.79)	< 0.001	0.97 (0.86, 1.08)	0.528	1.01 (0.91, 1.13)	0.858
Hispanic	0.53 (0.47, 0.58)	< 0.001	0.73 (0.65, 0.82)	< 0.001	0.53 (0.47, 0.59)	< 0.001
Non-Hispanic Other Races	0.59 (0.52, 0.66)	< 0.001	0.59 (0.49, 0.71)	< 0.001	0.68 (0.59, 0.77)	< 0.001
Annual income level						
Less than $75,000	Reference		Reference		Reference	
$75,000 or more	0.50 (0.46, 0.53)	< 0.001	0.95 (0.88, 1.03)	0.223	0.45 (0.41, 0.50)	< 0.001
Living in a state with a medical cannabis law						
No	Reference		Reference		Reference	
Yes	0.76 (0.71, 0.82)	< 0.001	1.84 (1.68, 2.02)	< 0.001	1.30 (1.17, 1.43)	< 0.001

Key: aRRR = adjusted Relative Risk Ratio; CI = confidence interval; NSDUH = National Survey on Drug Use and Health

Tables 3, 4, 5, 6 and 7 present the results of the main effects and interaction effects of HIV status and sociodemographic factors on past-month tobacco and cannabis co-use. Table 3 shows the results for the interaction between HIV status and age group, which was not significant (*p* = 0.3007). Table 4 presents the interaction between HIV status and sex, which was also not significant (*p* = 0.148). Table 5 shows a significant interaction between HIV status and race/ethnicity (*p* < 0.001). Linear combinations of coefficients of main effects and interaction effects showed that non-Hispanic Black adults with HIV had a higher likelihood of tobacco and cannabis co-use (aRRR = 5.02, 95% CI: 1.77–14.22, *p* = 0.003), while no significant difference was observed for Hispanic adults with HIV (*p* = 0.781) compared to non-Hispanic White adults without HIV. Table 6 presents the interaction between HIV status and annual income level, which was not statistically significant (*p* = 0.052). Table 7 shows a significant interaction between HIV status and state-level medical cannabis legalization status (*p* = 0.045). Linear combinations of coefficients of main effects and interaction effects showed that adults with HIV living in states with legalized medical cannabis had a higher likelihood of tobacco and cannabis co-use (aRRR = 2.60, 95% CI: 1.20–5.66) compared to adults without HIV living in states without legalized medical cannabis.

**Table 3:** Results of adjusted multinomial logistic regression on tobacco and cannabis co-use by lifetime HIV diagnosis status, age and HIV x age group, among adults from 2021–2023 NSDUH

	Tobacco only use		Cannabis only use		Co-use of tobacco and cannabis	
	aRRR (95% CI)	p-value	aRRR (95% CI)	p-value	aRRR (95% CI)	p-value
Lifetime HIV diagnosis status						
No	Reference		Reference		Reference	
Yes	1.84 (1.05, 3.25)	0.034	3.98 (2.24, 7.08)	< 0.001	3.23 (1.67, 6.21)	0.001
Age, years						
26 or older	Reference		Reference		Reference	
18–25	0.92 (0.86, 0.98)	0.016	1.74 (1.64, 1.86)	< 0.001	2.41 (2.22, 2.62)	< 0.001
HIV status x Age						Overall *p* = 0.2997
Yes x 18–25 years	1.96 (0.54, 7.11)	0.299	1.03 (0.32, 3.31)	0.961	3.05 (0.77, 12.14)	0.111
Survey years						
2021	Reference		Reference		Reference	
2022	0.98 (0.91, 1.06)	0.574	1.20 (1.07, 1.36)	0.003	1.16 (1.07, 1.26)	< 0.001
2023	1.00 (0.93, 1.08)	0.989	1.31 (1.17, 1.47)	< 0.001	1.20 (1.09, 1.33)	0.001
Sex						
Female	Reference		Reference		Reference	
Male	1.77 (1.68, 1.88)	< 0.001	1.34 (1.23, 1.44)	< 0.001	2.07 (1.95, 2.20)	< 0.001
Race/ethnicity						
Non-Hispanic White	Reference		Reference		Reference	
Non-Hispanic Black	0.73 (0.68, 0.79)	< 0.001	0.96 (0.86, 1.08)	0.526	1.01 (0.91, 1.13)	0.851
Hispanic	0.53 (0.47, 0.58)	< 0.001	0.73 (0.65, 0.82)	< 0.001	0.53 (0.47, 0.59)	< 0.001
Non-Hispanic Other Races	0.59 (0.52, 0.66)	< 0.001	0.59 (0.49, 0.71)	< 0.001	0.68 (0.59, 0.77)	< 0.001
Annual income level						
Less than $75,000	Reference		Reference		Reference	
$75,000 or more	0.50 (0.46, 0.53)	< 0.001	0.95 (0.88, 1.03)	0.223	0.45 (0.41, 0.50)	< 0.001
Living in a state with a medical cannabis law						
No	Reference		Reference		Reference	
Yes	0.76 (0.71, 0.82)	< 0.001	1.84 (1.68, 2.02)	< 0.001	1.30 (1.17, 1.43)	< 0.001

Key: aRRR = adjusted Relative Risk Ratio; CI = confidence interval; NSDUH = National Survey on Drug Use and Health

**Table 4:** Results of adjusted multinomial logistic regression on tobacco and cannabis co-use by lifetime HIV diagnosis status, sex and HIV x sex, among adults from 2021–2023 NSDUH

	Tobacco only use		Cannabis only use		Co-use of tobacco and cannabis	
	aRRR (95% CI)	p-value	aRRR (95% CI)	p-value	aRRR (95% CI)	p-value
Lifetime HIV diagnosis status						
No	Reference		Reference		Reference	
Yes	2.69 (1.09, 6.64)	0.033	2.06 (0.62, 6.84)	0.231	1.32 (0.51, 3.44)	0.559
Sex						
Female	Reference		Reference		Reference	
Male	1.78 (1.68, 1.88)	< 0.001	1.33 (1.23, 1.44)	< 0.001	2.06 (1.94, 2.19)	< 0.001
HIV status x Sex						Overall *p* = 0.1484
Yes x Male	0.65 (0.23, 1.84)	0.413	2.12 (0.55, 8.11)	0.266	2.79 (0.83, 9.34)	0.094
Survey years						
2021	Reference		Reference		Reference	
2022	0.98 (0.91, 1.06)	0.574	1.20 (1.07, 1.36)	0.003	1.16 (1.07, 1.26)	< 0.001
2023	1.00 (0.93, 1.08)	0.990	1.31 (1.17, 1.47)	< 0.001	1.20 (1.09, 1.33)	0.001
Age, years						
26 or older	Reference		Reference		Reference	
18–25	0.92 (0.86, 0.98)	0.016	1.74 (1.64, 1.86)	< 0.001	2.41 (2.23, 2.62)	< 0.001
Race/ethnicity						
Non-Hispanic White	Reference		Reference		Reference	
Non-Hispanic Black	0.73 (0.68, 0.79)	< 0.001	0.97 (0.86, 1.08)	0.540	1.01 (0.91, 1.13)	0.842
Hispanic	0.53 (0.47, 0.58)	< 0.001	0.73 (0.65, 0.82)	< 0.001	0.53 (0.47, 0.59)	< 0.001
Non-Hispanic Other Races	0.59 (0.52, 0.66)	< 0.001	0.59 (0.49, 0.71)	< 0.001	0.68 (0.59, 0.78)	< 0.001
Annual income level						
Less than $75,000	Reference		Reference		Reference	
$75,000 or more	0.50 (0.46, 0.53)	< 0.001	0.95 (0.88, 1.03)	0.222	0.45 (0.41, 0.50)	< 0.001
Living in a state with a medical cannabis law						
No	Reference		Reference		Reference	
Yes	0.76 (0.71, 0.82)	< 0.001	1.84 (1.68, 2.02)	< 0.001	1.30 (1.17, 1.43)	< 0.001

Key: aRRR = adjusted Relative Risk Ratio; CI = confidence interval; NSDUH = National Survey on Drug Use and Health

**Table 5:** Results of adjusted multinomial logistic regression on tobacco and cannabis co-use by lifetime HIV diagnosis status, race/ethnicity and HIV x race/ethnicity, among adults from 2021–2023 NSDUH

	Tobacco only use		Cannabis only use		Co-use of tobacco and cannabis	
	aRRR (95% CI)	p-value	aRRR (95% CI)	p-value	aRRR (95% CI)	p-value
Lifetime HIV diagnosis status						
No	Reference		Reference		Reference	
Yes	1.70 (0.88, 3.30)	0.112	4.46 (2.08, 9.54)	< 0.001	2.72 (0.96, 7.72)	0.059
Race/ethnicity						
Non-Hispanic White	Reference		Reference		Reference	
Non-Hispanic Black	0.73 (0.68, 0.79)	< 0.001	0.97 (0.87, 1.08)	0.558	1.00 (0.90, 1.12)	0.985
Hispanic	0.52 (0.47, 0.58)	< 0.001	0.73 (0.65, 0.81)	< 0.001	0.53 (0.47, 0.59)	< 0.001
Non-Hispanic Other Races	0.58 (0.52, 0.66)	< 0.001	0.59 (0.49, 0.71)	< 0.001	0.67 (0.59, 0.77)	< 0.001
HIV status x race/ethnicity						Overall *P* < 0.001
Yes x non-Hispanic Black	1.02 (0.40, 2.61)	0.963	0.84 (0.23, 3.03)	0.783	1.84 (0.45, 7.60)	0.392
Yes x Hispanic	1.28 (0.49, 3.35)	0.612	0.81 (0.21, 3.10)	0.753	0.81 (0.19, 3.48)	0.768
Yes x non-Hispanic Other Races	2.30 (0.41, 12.94)	0.339	- ^a^	- ^a^	2.94 (0.61, 14.30)	0.177
Survey years						
2021	Reference		Reference		Reference	
2022	0.98 (0.91, 1.06)	0.576	1.20 (1.07, 1.36)	0.003	1.16 (1.07, 1.26)	< 0.001
2023	1.00 (0.93, 1.08)	0.990	1.31 (1.17, 1.47)	< 0.001	1.20 (1.09, 1.33)	< 0.001
Age, years						
26 or older	Reference		Reference		Reference	
18–25	0.92 (0.86, 0.98)	0.017	1.74 (1.64, 1.86)	< 0.001	2.41 (2.22, 2.62)	< 0.001
Sex						
Female	Reference		Reference		Reference	
Male	1.77 (1.68, 1.88)	< 0.001	1.34 (1.23, 1.44)	< 0.001	2.07 (1.95, 2.20)	< 0.001
Annual income level						
Less than $75,000	Reference		Reference		Reference	
$75,000 or more	0.50 (0.46, 0.53)	< 0.001	0.95 (0.88, 1.03)	0.224	0.45 (0.41, 0.50)	< 0.001
Living in a state with a medical cannabis law						
No	Reference		Reference		Reference	
Yes	0.76 (0.71, 0.82)	< 0.001	1.84 (1.68, 2.03)	< 0.001	1.30 (1.17, 1.43)	< 0.001

Key: aRRR = adjusted Relative Risk Ratio; CI = confidence interval; NSDUH = National Survey on Drug Use and Health

a: not estimated due to small cell size

Note: Using linear combinations of coefficient parameters of main effects and an interaction effect, a higher level of tobacco and cannabis co-use was reported by non-Hispanic Black individuals with HIV (aRRR = 5.02, 95% CI = 1.77, 14.22, *p* = 0.003), but not by Hispanic individuals with HIV (*p* = 0.781), compared to non-Hispanic White individuals without HIV.

**Table 6:** Results of adjusted multinomial logistic regression on tobacco and cannabis co-use by lifetime HIV diagnosis status, income level and HIV x income level, among adults from 2021–2023 NSDUH

	Tobacco only use		Cannabis only use		Co-use of tobacco and cannabis	
	aRRR (95% CI)	p-value	aRRR (95% CI)	p-value	aRRR (95% CI)	p-value
Lifetime HIV diagnosis status						
No	Reference		Reference		Reference	
Yes	2.97 (1.14, 7.78)	0.027	6.44 (2.63, 15.75)	< 0.001	11.89 (4.14, 34.21)	< 0.001
Annual income level						
Less than $75,000	2.01 (1.88, 2.15)	< 0.001	1.05 (0.97, 1.14)	0.212	2.23 (2.03, 2.45)	< 0.001
$75,000 or more	Reference		Reference		Reference	
HIV status x income level						Overall *P* = 0.052
Yes x less than $75,000	0.55 (0.19, 1.62)	0.275	0.54 (0.20, 1.47)	0.224	0.19 (0.06, 0.64)	0.008
Survey years						
2021	Reference		Reference		Reference	
2022	0.98 (0.91, 1.06)	0.576	1.20 (1.07, 1.36)	0.003	1.16 (1.07, 1.26)	< 0.001
2023	1.00 (0.93, 1.08)	0.986	1.31 (1.17, 1.48)	< 0.001	1.20 (1.09, 1.33)	0.001
Age, years						
26 or older	Reference		Reference		Reference	
18–25	0.92 (0.86, 0.98)	0.017	1.74 (1.64, 1.86)	< 0.001	2.41 (2.22, 2.62)	< 0.001
Sex						
Female	Reference		Reference		Reference	
Male	1.77 (1.68, 1.88)	< 0.001	1.34 (1.23, 1.44)	< 0.001	2.07 (1.95, 2.20)	< 0.001
Race/ethnicity						
Non-Hispanic White	Reference		Reference		Reference	
Non-Hispanic Black	0.73 (0.68, 0.79)	< 0.001	0.97 (0.86, 1.08)	0.530	1.01 (0.91, 1.13)	0.832
Hispanic	0.53 (0.47, 0.58)	< 0.001	0.73 (0.65, 0.82)	< 0.001	0.53 (0.47, 0.59)	< 0.001
Non-Hispanic Other Races	0.59 (0.52, 0.66)	< 0.001	0.59 (0.49, 0.71)	< 0.001	0.68 (0.59, 0.77)	< 0.001
Living in a state with a medical cannabis law						
No	Reference		Reference		Reference	
Yes	0.76 (0.71, 0.82)	< 0.001	1.84 (1.68, 2.03)	< 0.001	1.30 (1.17, 1.43)	< 0.001

Key: aRRR = adjusted Relative Risk Ratio; CI = confidence interval

**Table 7:** Results of adjusted multinomial logistic regression on tobacco and cannabis co-use by lifetime HIV diagnosis status, living in States that legalized medical cannabis law and HIV x living in States that legalized medical cannabis law, among adults from 2021–2023 NSDUH

	Tobacco only use		Cannabis only use		Co-use of tobacco and cannabis	
	aRRR (95% CI)	p-value	aRRR (95% CI)	p-value	aRRR (95% CI)	p-value
Lifetime HIV diagnosis status						
No	Reference		Reference		Reference	
Yes	3.81 (1.12, 12.97)	0.033	5.88 (1.92, 17.94)	0.002	13.99 (4.85, 40.37)	< 0.001
Living in a state with a medical cannabis law						
No	Reference		Reference		Reference	
Yes	0.76 (0.71, 0.82)	< 0.001	1.84 (1.68, 2.02)	< 0.001	1.31 (1.19, 1.45)	< 0.001
HIV status x living in a state with a medical cannabis law						Overall *P* = 0.0445
Yes x Yes	0.41 (0.09, 1.81)	0.232	0.61 (0.18, 2.02)	0.406	0.14 (0.04, 0.55)	0.005
Survey years						
2021	Reference		Reference		Reference	
2022	0.98 (0.91, 1.06)	0.574	1.20 (1.07, 1.36)	0.003	1.16 (1.07, 1.26)	< 0.001
2023	1.00 (0.93, 1.08)	0.993	1.31 (1.17, 1.47)	< 0.001	1.20 (1.09, 1.33)	0.001
Age, years						
26 or older	Reference		Reference		Reference	
18–25	0.92 (0.86, 0.98)	0.017	1.74 (1.64, 1.86)	< 0.001	2.41 (2.23, 2.62)	< 0.001
Sex						
Female	Reference		Reference		Reference	
Male	1.78 (1.68, 1.88)	< 0.001	1.34 (1.23, 1.44)	< 0.001	2.07 (1.95, 2.20)	< 0.001
Race/ethnicity						
Non-Hispanic White	Reference		Reference		Reference	
Non-Hispanic Black	0.73 (0.68, 0.79)	< 0.001	0.97 (0.86, 1.08)	0.528	1.01 (0.90, 1.12)	0.919
Hispanic	0.53 (0.47, 0.58)	< 0.001	0.73 (0.65, 0.82)	< 0.001	0.53 (0.47, 0.59)	< 0.001
Non-Hispanic Other Races	0.59 (0.52, 0.66)	< 0.001	0.59 (0.49, 0.71)	< 0.001	0.67 (0.59, 0.77)	< 0.001
Annual income level						
Less than $75,000	Reference		Reference		Reference	
$75,000 or more	0.50 (0.46, 0.53)	< 0.001	0.95 (0.88, 1.03)	0.223	0.45 (0.41, 0.50)	< 0.001

Note: Using linear combinations of coefficient parameters of main effects and an interaction effect, a higher level of tobacco and cannabis co-use was reported by US adults with HIV living in states that legalized medical cannabis law (aRRR = 2.60, 95% CI = 1.20, 5.66, *p* = 0.017), compared to US adults without HIV not living in states that legalized medical cannabis law

## Discussion

This study provides novel information about tobacco and cannabis co-use prevalence, overall and by sociodemographic variables, among people with HIV. Compared to U.S. adults without HIV, U.S. adults with HIV reported higher levels of tobacco and cannabis co-use, with an estimated 178,775 U.S. adults with HIV engaged in co-use. The elevated levels of tobacco and cannabis co-use in individuals with HIV remained significant across multiple sensitivity analyses, adjusting for additional covariates. The higher prevalence of co-use may be driven by the use of tobacco and cannabis to relieve stress associated with a diagnosis of HIV or for self-medication, such as pain and symptom management [23, 38]. Alternatively, individuals with HIV may also use tobacco and cannabis recreationally, similar to the general population [23, 39]. Future research should examine the underlying reasons and contextual factors (e.g., who, where, and when) associated with tobacco and cannabis co-use. Such insights would help inform tailored prevention and cessation strategies, including educational programs and public awareness campaigns, targeted at individuals with HIV.

We also examined sociodemographic differences in tobacco and cannabis co-use patterns among people with HIV. This study found significant interaction effects between HIV status and race/ethnicity in relation to tobacco and cannabis co-use, suggesting that non-Hispanic Black adults with HIV may be at increased risk for co-use. We speculate that this increased risk may result from targeted tobacco and cannabis marketing toward non-Hispanic Black communities, a pattern observed in the general population [40–42]. Additionally, non-Hispanic Black adults with HIV may experience heightened levels of stress due to racial and ethnic discrimination, compounded by greater HIV-related stigma compared to non-Hispanic White adults with HIV [43–45]. These findings are concerning, as they may contribute to worsening HIV-related health disparities and tobacco- and cannabis-associated health risks in this population. Given that non-Hispanic Black adults are already disproportionately affected by HIV [24], there is an urgent need to investigate the underlying factors driving this elevated co-use risk and to develop tailored clinical interventions and educational programs to address it.

This study also found a significant interaction between HIV status and state-level medical cannabis legalization status, indicating that U.S. adults with HIV living in states with medical cannabis laws may be at higher risk for tobacco and cannabis co-use. This finding is expected given that high levels of co-use of tobacco and cannabis are reported among the general population residing in states with legalized medical cannabis [1, 26, 46]. However, a study by Pravosud et al. found that while tobacco and cannabis co-use increased between 2017 and 2021 among adults in the U.S., it was not significantly associated with state-level medical or recreational cannabis legalization [47]. Although medical cannabis is recognized for its potential benefits in managing HIV-related inflammation and symptoms [14, 48], the combined effects of tobacco and cannabis co-use remain uncertain and may pose additional health risks for people with HIV. Further research is needed to clarify these potential harms and to inform evidence-based harm reduction strategies.

Notably, state-level medical cannabis legalization status attenuated the magnitude of the effects of HIV diagnosis on the risk of tobacco and cannabis co-use. One speculation is that living in a state with medical cannabis law might be related to increased exclusive cannabis and reduced level of tobacco use (i.e., a substitution effect). For example, a study by Dave et al. (2023) used two U.S. national datasets and observed that adoption of recreational cannabis laws were associated with increased levels of cannabis use, but decreased levels of tobacco use among adults [49]. Another study by De and Sun (2025) used the 2021–2022 Behavioral Risk Factor Surveillance System and observed that recreational cannabis laws were associated with current cigarette use [50]. A systematic review by Farrelly et al. (2023) found that recreational cannabis laws were associated with increased levels of cannabis use, in general, and decreasing rates of cigarette smoking, suggesting a potential substitution effect [51]. Even though these studies were specifically focused on recreational cannabis laws, we speculate that this substitution effect might affect the attenuated risk ratio of tobacco and cannabis co-use when considering HIV status. Another previous study by Weinberger and colleagues found that medical cannabis legalization was associated with an increase in cigarette and cannabis co-use, but no change in cigarette-only use or cannabis-only use among the overall U.S. population [26]. However, medical cannabis legalization was associated with increased levels in cigarette-only use and cannabis-only use among those aged 50 years and older, suggesting that there might be complex interactions between living in states with cannabis-related laws and other factors such as sociodemographics. Future studies should examine the complex interplay between cannabis-related laws, HIV status, and other factors.

This study provides novel insights into the prevalence of tobacco and cannabis co-use among people with HIV, highlighting subgroup differences based on sociodemographic factors, with important public health and clinical implications. A key strength of this study is the use of a nationally representative U.S. sample, which enhances generalizability and enables population-level estimates. Further, the findings are robust, supported by multiple sensitivity analyses incorporating additional covariates. However, this study has several limitations. First, data were self-reported, which may introduce biases, including recall bias and social desirability bias, particularly regarding HIV status and cannabis use behaviors. Second, the NSDUH survey includes only U.S. civilian, non-institutionalized individuals, excluding populations with higher levels of substance use and HIV vulnerability, such as incarcerated individuals and those experiencing homelessness [52, 53]. Third, the NSDUH does not collect detailed data on the frequency and dosage of tobacco and cannabis use, including nicotine concentration, THC concentration, and reasons for cannabis use (e.g., medicinal versus recreational cannabis use), nor does it distinguish between concurrent, simultaneous, or sequential use of tobacco and cannabis [54]. Fourth, unmeasured confounders may influence the observed associations, such as state-level recreational cannabis legalization status and exposure to tobacco and cannabis marketing. Fifth, even though we combined several years of US national datasets, the sample size of adults with HIV who reported past-month tobacco and cannabis co-use was still small enough that we were unable to examine the potential range of product combinations related to tobacco and cannabis co-use by HIV status (e.g., cigarette use versus vaping nicotine, vaping cannabis versus sublingual cannabis use). Future studies should employ a larger-scale HIV-focused dataset and examine the various co-use patterns among people with HIV.

Sixth, the public use NSDUH data only indicated whether respondents were living in a state that had passed a law allowing the use of cannabis for medical reasons at the time of the interview and more specific information about these medical cannabis laws was not available. For example, some states only allow low-THC products while others allow full cannabis use. Further, some states have restrictive laws that only permit CBD or require specific conditions (e.g., policy variation across states). Further, even though we used residence in a state with a medical cannabis law as a proxy for policy exposure, this measure may not capture individual-level awareness, eligibility, or access to medical cannabis programs, and such misclassification may affect observed effect modification. In addition, the residence in states with medical cannabis laws might not fully capture residence stability as respondents may move among states with different cannabis-related laws over time. Future studies should examine more detailed aspects of cannabis laws in relation to tobacco and cannabis co-use among people with HIV.

## Conclusions

This study highlights the greater prevalence of tobacco and cannabis co-use among people with HIV, particularly among non-Hispanic Black adults with HIV and adults with HIV residing in states with legalized medical cannabis. These findings inform future research directions, emphasizing the need to explore motivations for co-use among people with HIV. Further understanding of these factors will support the development of targeted prevention and cessation strategies, such as integrating patient education on the health risks of tobacco and cannabis co-use into clinical practice and implementing public awareness programs to reduce co-use in this population. Additionally, policy-level awareness and interventions should address the unintended consequences of cannabis legalization, particularly the potential for increased co-use of tobacco and cannabis among people with HIV.

## Electronic supplementary material

Below is the link to the electronic supplementary material.

Supplementary Material 1

## Abbreviations

HIV: Human Immunodeficiency Virus
US: United States
NSDUH: National Survey on Drug Use and Health
STROBE: Strengthening the Reporting of Observational Studies in Epidemiology
aRRR: Adjusted Relative Risk Ratio
CI: Confidence interval
