# Supplementary material for: Tobacco and Cannabis Co-use by HIV Status Among United States Adults: Results from the 2021–2023 National Survey on Drug Use and Health
Source: AIDS Behav. 2025 Jul 4;29(12):3794–805. doi: 10.1007/s10461-025-04817-5 (PMC12580427; doi:10.1007/s10461-025-04817-5)
Supplement: Supplementary file 1 — Supplementary Material 1 [file 10461_2025_4817_MOESM1_ESM.docx]

**Supplemental Table 1**. Results of adjusted multinomial logistic regression on tobacco and cannabis co-use by lifetime HIV diagnosis status among adults from 2021-2023 NSDUH, with additional covariate “sexual identity”

|  | **Tobacco only use** |  | **Cannabis only use** |  | **Co-use of**  **tobacco and cannabis** |  |
| --- | --- | --- | --- | --- | --- | --- |
|  | aRRR (95% CI) | p-value | aRRR (95% CI) | p-value | aRRR (95% CI) | p-value |
| **Lifetime HIV diagnosis status** |  |  |  |  |  |  |
| No | Reference |  | Reference |  | Reference |  |
| Yes | 1.44 (0.80, 2.58) | 0.222 | 1.82 (0.94, 3.52) | 0.073 | **2.10 (1.09, 4.04)** | **0.027** |
| **Survey years** |  |  |  |  |  |  |
| 2021 | Reference |  | Reference |  | Reference |  |
| 2022 | 0.97 (0.90, 1.05) | 0.463 | 1.18 (1.05, 1.33) | 0.005 | 1.14 (1.05, 1.24) | 0.002 |
| 2023 | 1.00 (0.93, 1.09) | 0.947 | 1.30 (1.16, 1.47) | <0.001 | 1.22 (1.11, 1.35) | <0.001 |
| **Age, years** |  |  |  |  |  |  |
| 26 or older | Reference |  | Reference |  | Reference |  |
| 18-25 | 0.88 (0.82, 0.94) | <0.001 | 1.40 (1.30, 1.51) | <0.001 | 1.97 (1.80, 2.16) | <0.001 |
| **Sex** |  |  |  |  |  |  |
| Female | Reference |  | Reference |  | Reference |  |
| Male | 1.81 (1.71, 1.93) | <0.001 | 1.46 (1.35, 1.57) | <0.001 | 2.32 (2.18, 2.49) | <0.001 |
| **Race/ethnicity** |  |  |  |  |  |  |
| Non-Hispanic White | Reference |  | Reference |  | Reference |  |
| Non-Hispanic Black | 0.74 (0.68, 0.80) | <0.001 | 1.00 (0.89, 1.13) | 0.979 | 1.07 (0.96, 1.20) | 0.217 |
| Hispanic | 0.52 (0.47, 0.58) | <0.001 | 0.74 (0.66, 0.84) | <0.001 | 0.55 (0.49, 0.62) | <0.001 |
| Non-Hispanic Other Races | 0.59 (0.53, 0.66) | <0.001 | 0.62 (0.51, 0.75) | <0.001 | 0.70 (0.61, 0.81) | <0.001 |
| **Annual income level** |  |  |  |  |  |  |
| Less than $75,000 | Reference |  | Reference |  | Reference |  |
| $75,000 or more | 0.49 (0.46, 0.53) | <0.001 | 0.97 (0.88, 1.05) | 0.427 | 0.46 (0.42, 0.51) | <0.001 |
| **Living in states that legalized medical cannabis law** |  |  |  |  |  |  |
| No | Reference |  | Reference |  | Reference |  |
| Yes | 0.77 (0.71, 0.82) | <0.001 | 1.89 (1.71, 2.08) | <0.001 | 1.30 (1.18, 1.44) | <0.001 |
| **Sexual identity** |  |  |  |  |  |  |
| Heterosexual/straight | Reference |  | Reference |  | Reference |  |
| Gay/lesbian | 1.23 (1.07, 1.43) | 0.005 | 2.77 (2.28, 3.37) | <0.001 | 1.76 (1.47, 2.10) | <0.001 |
| Bisexual | 1.75 (1.57, 1.95) | <0.001 | 4.11 (3.64, 4.63) | <0.001 | 4.35 (3.95, 4.80) | <0.001 |

**Supplemental Table 2**. Results of adjusted multinomial logistic regression on tobacco and cannabis co-use by lifetime HIV diagnosis status among adults from 2021-2023 NSDUH, with additional covariate “past-month other substance use and misuse”

|  | **Tobacco only use** |  | **Cannabis only use** |  | **Co-use of**  **tobacco and cannabis** |  |
| --- | --- | --- | --- | --- | --- | --- |
|  | aRRR (95% CI) | p-value | aRRR (95% CI) | p-value | aRRR (95% CI) | p-value |
| **Lifetime HIV diagnosis status** |  |  |  |  |  |  |
| No | Reference |  | Reference |  | Reference |  |
| Yes | 1.79 (1.02, 3.15) | 0.043 | 3.65 (2.05, 6.48) | <0.001 | **3.02 (1.60, 5.69)** | **0.001** |
| **Survey years** |  |  |  |  |  |  |
| 2021 | Reference |  | Reference |  | Reference |  |
| 2022 | 0.98 (0.90, 1.05) | 0.523 | 1.20 (1.06, 1.35) | 0.004 | 1.16 (1.07, 1.26) | 0.001 |
| 2023 | 1.01 (0.93, 1.09) | 0.879 | 1.33 (1.18, 1.50) | <0.001 | 1.22 (1.10, 1.35) | <0.001 |
| **Age, years** |  |  |  |  |  |  |
| 26 or older | Reference |  | Reference |  | Reference |  |
| 18-25 | 0.94 (0.87, 1.00) | 0.054 | 1.83 (1.72, 1.95) | <0.001 | 2.51 (2.31, 2.73) | <0.001 |
| **Sex** |  |  |  |  |  |  |
| Female | Reference |  | Reference |  | Reference |  |
| Male | 1.74 (1.64, 1.85) | <0.001 | 1.30 (1.20, 1.40) | <0.001 | 2.01 (1.89, 2.14) | <0.001 |
| **Race/ethnicity** |  |  |  |  |  |  |
| Non-Hispanic White | Reference |  | Reference |  | Reference |  |
| Non-Hispanic Black | 0.76 (0.70, 0.82) | <0.001 | 1.06 (0.94, 1.19) | 0.353 | 1.11 (1.00, 1.24) | 0.050 |
| Hispanic | 0.53 (0.48, 0.59) | <0.001 | 0.77 (0.68, 0.86) | <0.001 | 0.56 (0.49, 0.63) | <0.001 |
| Non-Hispanic Other Races | 0.63 (0.56, 0.71) | <0.001 | 0.71 (0.59, 0.86) | 0.001 | 0.81 (0.70, 0.94) | 0.006 |
| **Annual income level** |  |  |  |  |  |  |
| Less than $75,000 | Reference |  | Reference |  | Reference |  |
| $75,000 or more | 0.45 (0.42, 0.48) | <0.001 | 0.76 (0.70, 0.83) | <0.001 | 0.36 (0.33, 0.39) | <0.001 |
| **Living in states that legalized medical cannabis law** |  |  |  |  |  |  |
| No | Reference |  | Reference |  | Reference |  |
| Yes | 0.76 (0.71, 0.82) | <0.001 | 1.83 (1.67, 2.01) | <0.001 | 1.29 (1.16, 1.43) | <0.001 |
| **Past-month other substance use other than tobacco and cannabis** |  |  |  |  |  |  |
| None | Reference |  | Reference |  | Reference |  |
| Any | 1.67 (1.55, 1.81) | <0.001 | 3.63 (3.35, 3.92) | <0.001 | 4.00 (3.56, 4.49) | <0.001 |

“Past-month other substance use other than tobacco and cannabis” include alcohol, cocaine/crack, heroin, hallucinogens, inhalants, methamphetamine, misuses of pain relievers, tranquilizers, stimulants, sedatives.

**Supplemental Table 3**. Results of adjusted multinomial logistic regression on tobacco and cannabis co-use by lifetime HIV diagnosis status among adults from 2021-2023 NSDUH, with additional covariate “past-month serious psychological distress”

|  | **Tobacco only use** |  | **Cannabis only use** |  | **Co-use of**  **tobacco and cannabis** |  |
| --- | --- | --- | --- | --- | --- | --- |
|  | aRRR (95% CI) | p-value | aRRR (95% CI) | p-value | aRRR (95% CI) | p-value |
| **Lifetime HIV diagnosis status** |  |  |  |  |  |  |
| No | Reference |  | Reference |  | Reference |  |
| Yes | 1.87 (1.06, 3.29) | 0.031 | 3.96 (2.25, 6.95) | <0.001 | **3.33 (1.81, 6.13)** | **<0.001** |
| **Survey years** |  |  |  |  |  |  |
| 2021 | Reference |  | Reference |  | Reference |  |
| 2022 | 0.98 (0.90, 1.05) | 0.521 | 1.20 (1.07, 1.35) | 0.003 | 1.15 (1.06, 1.25) | 0.001 |
| 2023 | 1.00 (0.93, 1.08) | 0.968 | 1.31 (1.16, 1.47) | <0.001 | 1.20 (1.08, 1.33) | 0.001 |
| **Age, years** |  |  |  |  |  |  |
| 26 or older | Reference |  | Reference |  | Reference |  |
| 18-25 | 0.85 (0.80, 0.91) | <0.001 | 1.56 (1.47, 1.67) | <0.001 | 2.02 (1.86, 2.20) | <0.001 |
| **Sex** |  |  |  |  |  |  |
| Female | Reference |  | Reference |  | Reference |  |
| Male | 1.81 (1.71, 1.91) | <0.001 | 1.37 (1.27, 1.48) | <0.001 | 2.19 (2.05, 2.33) | <0.001 |
| **Race/ethnicity** |  |  |  |  |  |  |
| Non-Hispanic White | Reference |  | Reference |  | Reference |  |
| Non-Hispanic Black | 0.74 (0.68, 0.79) | <0.001 | 0.97 (0.87, 1.09) | 0.588 | 1.02 (0.91, 1.15) | 0.713 |
| Hispanic | 0.53 (0.48, 0.59) | <0.001 | 0.73 (0.65, 0.82) | <0.001 | 0.53 (0.48, 0.60) | <0.001 |
| Non-Hispanic Other Races | 0.58 (0.52, 0.65) | <0.001 | 0.58 (0.48, 0.70) | <0.001 | 0.66 (0.58, 0.76) | <0.001 |
| **Annual income level** |  |  |  |  |  |  |
| Less than $75,000 | Reference |  | Reference |  | Reference |  |
| $75,000 or more | 0.51 (0.48, 0.54) | <0.001 | 0.98 (0.90, 1.07) | 0.649 | 0.48 (0.44, 0.53) | <0.001 |
| **Living in states that legalized medical cannabis law** |  |  |  |  |  |  |
| No | Reference |  | Reference |  | Reference |  |
| Yes | 0.77 (0.71, 0.82) | <0.001 | 1.85 (1.69, 2.03) | <0.001 | 1.31 (1.18, 1.44) | <0.001 |
| **Past-month serious psychological distress** |  |  |  |  |  |  |
| None | Reference |  | Reference |  | Reference |  |
| Any | 1.98 (1.80, 2.17) | <0.001 | 2.36 (2.15, 2.59) | <0.001 | 3.70 (3.40, 4.04) | <0.001 |
